# Supplementary material for: Identification of Freezing-Responsive microRNAs and Their Targets in Chinese Jujube by Small RNA and Degradome Sequencing
Source: Int J Mol Sci. 2026 Sep 4;27(17):7912. doi: 10.3390/ijms27177912 (PMC13566890; doi:10.3390/ijms27177912)
Supplement: Supplementary file 1 [file ijms-27-07912-s001.zip › Supplementary Material S2/novel_miR_117.pdf]

Provisional ID	:	novel_miR_117
Score total	:	432.2
Score for star read(s)	:	3.9
Score for read counts	:	425.3
Score for mfe	:	1.4
Score for randfold	:	1.6
Score for cons. seed	:	
Total read count	:	846
Mature read count	:	822
Loop read count	:	0
Star read count	:	24

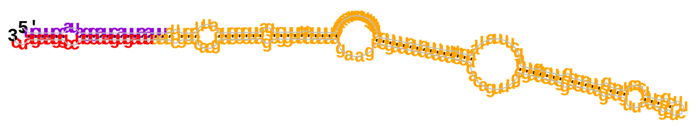

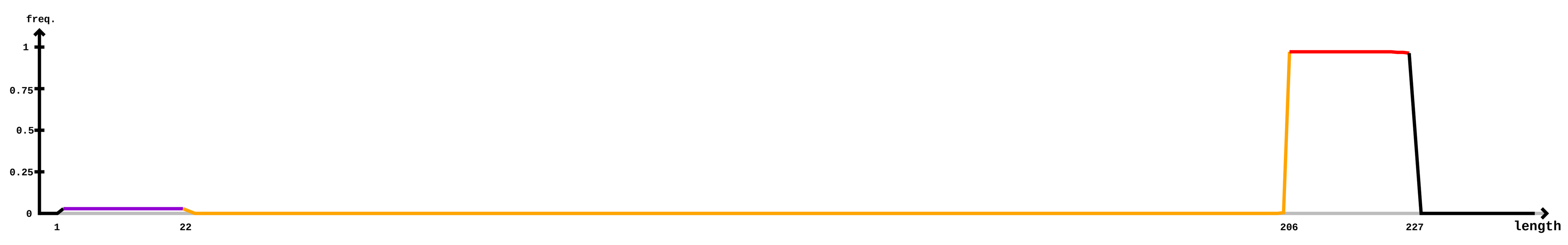

Star

Mature

```
5'- gaucuuccauaggaucauuaauuauugauuaaauggguuaggcuuguauuuuggaaaauuuauggguuagguuuauauuuuuggaauuauuucgaguugaguugggauuggauucacuuuggcuucuggcaauuguugaaucuggacuagcguuugacacuccaaaauauaagcugaagaaaauuaggcgaacccaugcacucgcuaauuagugauccuccggaagaucauaauccaguuuggaaccaauu -3'  obs
    gaucuuccauaggaucauuaauuauugauuaaauggguuaggcuuguauuuuggaaaauuuauggguuagguuuauauuuuuggaauuauuucgaguugaguugggauuggauucacuuuggcuucuggcaauuguugaaucuggacuagcguuugacacuccaaaauauaagcugaagaaaauuaggcgaacccaugcacucgcuaauuagugauccuccggaagaucauaauccaguuuggaaccaauu      exp
    (((((((..(((((((((((((.(((...(((((((..(((((.(((((................(((((((((((.(((..........(((.((((.(((((.(((.....(((.(....).)))..))).))))).))))))).........)))))))))))))))....))))))))))).)))))))....))).))))))))))))))..))))))))....((((...)))).......  reads  mm  sample
    ..ucuuccauaggaucauuaauu......................................................................................................................................................................................................  2    0   LT-3
    ..ucuuccauaggaucauuaauu......................................................................................................................................................................................................  4    0   JCK-2
    ..ucuuccauaggaucauuaauu......................................................................................................................................................................................................  1    0   JT-3
    ..ucuuccauaggaucauuaauu......................................................................................................................................................................................................  1    0   JT-2
    ..ucuuccauaggaucauuaauu......................................................................................................................................................................................................  6    0   JCK-1
    ..ucuuccauaggaucauuaauu......................................................................................................................................................................................................  10   0   JCK-3
    .......................................................................................................................................................................................auuagugauccuccggaag..........................  1    0   JCK-2
    .......................................................................................................................................................................................Cuuagugauccuccggaagauc.......................  2    1   JCK-1
    .......................................................................................................................................................................................Cuuagugauccuccggaagauc.......................  1    1   JCK-3
    ........................................................................................................................................................................................uuagugauccuccggaag..........................  1    0   JCK-1
    ........................................................................................................................................................................................uuagugauccuccggaag..........................  1    0   JCK-2
    ........................................................................................................................................................................................uuagugauccuccggaagUu........................  2    1   LT-2
    ........................................................................................................................................................................................uuagugauccuccggaagUu........................  1    1   JCK-1
    ........................................................................................................................................................................................uuagugauccuccggaagau........................  1    0   JCK-3
    ........................................................................................................................................................................................uuagugauccuccggaagauc.......................  23   0   JT-3
    ........................................................................................................................................................................................uuCgugauccuccggaagauc.......................  1    1   JT-2
    ........................................................................................................................................................................................uuagugauccuccggaagauU.......................  2    1   JCK-3
    ........................................................................................................................................................................................uuagugauccuccggaagauc.......................  21   0   JT-2
    ........................................................................................................................................................................................uuagugauccuccggaagauc.......................  12   0   LT-2
    ........................................................................................................................................................................................uuagugauccuccggaagauc.......................  177  0   JCK-1
    ........................................................................................................................................................................................uuCgugauccuccggaagauc.......................  1    1   JCK-2
    ........................................................................................................................................................................................uuagugauccuccggaagauc.......................  6    0   LT-3
    ........................................................................................................................................................................................uuagugauUcuccggaagauc.......................  1    1   JCK-2
    ........................................................................................................................................................................................uuagugauccuccggaCgauc.......................  1    1   JCK-2
    ........................................................................................................................................................................................uuagugauccuccggaagauU.......................  1    1   LT-3
    ........................................................................................................................................................................................uuagugauccuccggaagauc.......................  220  0   JCK-3
    ........................................................................................................................................................................................uuagugauccuccggaagCuc.......................  1    1   JCK-1
    ........................................................................................................................................................................................uuagugaucAuccggaagauc.......................  1    1   JCK-2
    ........................................................................................................................................................................................uuagugauccuccggaagauc.......................  326  0   JCK-2
    ........................................................................................................................................................................................uNagugauccuccggaagauc.......................  1    1   JCK-2
    ........................................................................................................................................................................................uuagugauccuccggaagauU.......................  1    1   JCK-2
    ........................................................................................................................................................................................uuagugauccuccggaagauc.......................  8    0   LCK-3
    ........................................................................................................................................................................................uuagugauccuccggaagauc.......................  8    0   LT-1
```

Star Mature

gaucuuccauaggaucauuaauuauugauuaauggguuaggcuuguauuuuggaaaauuuauggguuagguuuauauuuuuggaauuauuucgaguugaguugggauuggauucacuuuggcuucuggcaauuguugaaucuggacuagcguuugacacuccaaaauauaagcugaagaaaauuaggcgaacccaugcacucgcuaauuagugauccuccggaagaucauaauccaguuuggaaccaauu
